# The Clinical Efficacy and Safety of 11 Commonly Used Treatment Strategies Improving Arrhythmia of CHD in China: A Network Meta-Analysis

**DOI:** 10.3389/fphar.2021.741716

**Published:** 2021-09-20

**Authors:** Tao Wang, Wei Li, Qianqian Huang, Chuqiao Yuan, Liping Qu, Xiaohe Xiao, Wenjun Zou

**Affiliations:** ^1^ College of Pharmacy, Chengdu University of Traditional Chinese Medicine, Chengdu, China; ^2^ The Fifth Medical Center of PLA General Hospital, Beijing, China

**Keywords:** arrhythmia, clinical efficacy, safety, network meta-analysis, systematic review

## Abstract

**Purpose:** Arrhythmia which as a common complication of CHD, has a high incidence. At present, more and more anti-arrhythmic drugs are used in clinical practice. However, which drug has the best efficacy and high safety is still unknown. Therefore, we decided to use NMA to solve this problem.

**Method:** We searched CNKI, Wanfang database, VIP database, Pubmed, Embase and Cochrane libraries, and collected all RCTs of arrhythmia of CHD, and used RevMan (5.3) and Stata (13.0) to carry out this NMA. The primary outcome indicator of this study is efficiency; the secondary outcome indicator is the incidence of adverse reactions.

**Result:** A total of 134 RCTs, 13,951 patients, and 11 treatment strategies were included in this NMA. The results show that all treatment strategies can effectively improve the arrhythmia of patients. Among them, PMA+AM, AM+AT, AM+WG have higher effective rates, and PMA+AM, WG+ME, SC+ME have better safety. The effectiveness and safety of the treatment strategies which combined TCM and chemical drugs, are significantly better than that of using chemical drugs alone.

**Conclusion:** The treatment strategy of combination of multiple drugs usually has higher efficiency and safety. PMA+AM seems to be the most recommended treatment strategy. In addition, the rational combination of TCM and chemical drugs may provide potential benefit.

**Systematic Review Registration**: https://www.crd.york.ac.uk/PROSPERO/, identifier CRD42021229693.

## Introduction

Arrhythmias of coronary heart disease (CHD) are caused by the imbalance of myocardial electrical activity due to the stenosis or occlusion of the coronary lumen, and its incidence is as high as 40–75% (Kai-Qiang et al., 2019). In addition, the incidence of arrhythmia in elderly patients is higher, such as, the incidence of arrhythmia in people over 75 years old can be as high as 69% (Katta et al., 2020). Arrhythmia of CHD is mainly divided into sinus tachycardia, bradycardia, atrial or ventricular premature beats, supraventricular or ventricular tachycardia, ventricular fibrillation, and atrial fibrillation (Gladding et al., 2019). At present, the commonly used treatments for arrhythmia of CHD include: radiofrequency catheter ablation (RFCA), pacemaker implantation, and drug therapy (sodium channel blockers, β-adrenergic receptor blockers, calcium antagonists, etc.) (Zhang et al., 2019; Ye et al., 2021).

It has been reported that RFCA has a low success rate in areas such as ventricular, atrial, junctional premature beats and tachycardia (Zagkli et al., 2021), and pacemakers are expensive, and patients are afraid of implanting pacemakers (Klotzbaugh et al., 2020). However, drug therapy has become the most commonly used therapeutic strategy due to its efficacy and low treatment cost (Gaur et al., 2020). However, although antiarrhythmic drugs can reduce arrhythmia in patients through various ways, their effective rate is unstable and has different degrees of side effects, such as gastrointestinal toxicity, liver injury, cardiotoxicity, and allergic reactions. In addition, patients with structural heart disease have a higher risk of developing ventricular arrhythmias after using antiarrhythmic drugs (Shi, 2020). Therefore, it is particularly important to comprehensively evaluate the clinical efficacy and safety of commonly used clinical antiarrhythmic drugs to determine the most potential therapeutic strategy.

There are eight kinds of antiarrhythmic drugs which the most commonly used in China. Among them, Wenxin Granules (WG) and Shensong Yangxin Capsules (SC) are traditional Chinese medicines, while amiodarone (AM), metoprolol (ME), atenolol (AT), potassium magnesium aspartate (PMA), propafenone (PR) and lidocaine (LI) are chemical drugs. In this study, the network meta-analysis method was adopted to evaluate the clinical efficacy and safety of 11 treatment strategies including the above eight drugs, in order to provide reference for the clinical treatment of arrhythmia of CHD.

## Materials and Methods

### Search Strategy

This research protocol has been registered and approved on the international prospective register of systematic reviews (PROSPERO) on February 6, 2020. The registration number is: CRD42021229693. This study will be implemented in strict accordance with the registered research protocol. We searched China National Knowledge Infrastructure (CNKI), Wanfang Database, VIP medicine information system, PubMed, Embase and Cochrane Libraries over a period of time from the establishment of the database to February 2021. The initial search items were used as follows: “arrhythmia”, “coronary heart disease” (title/abstract) and “randomized controlled trial”(title/abstract). The inclusion criteria were as follows: 1) RCTs related to arrhythmia of CHD. 2) All patients were diagnosed as arrhythmia of CHD in accordance with clear diagnostic criteria. 3) The gender and age of patients were not limited. 4) The language of the literature is not limited. The exclusion criteria were as follows: 1) Repetitive published studies. 2) Studies with incomplete or incorrect data. 3) Animal experiments and literature review. In this study, three researchers (Tao Wang, Wei Li, and Qianqian Huang) independently extracted data. In addition, a review team of two researchers (Wenjun Zou, Liping Qu) checked the accuracy of the data and assessed the quality of the included studies. Basic information of the included studies was recorded (name of the study included, number of patients, therapeutic strategies, mode of administration, dose administered, course of treatment, outcome, etc.) and relevant data were extracted. The Cochrane Risk of Bias tool was used to assess the quality of the included studies. All the differences that occurred in the study were discussed by review team.

### Statistical Analysis

We performed this study using RevMan (5.3) and Stata (13.0) and constructed a treatment strategy network. For the dichotomous variable, we calculated the odds ratios (ORs), and all of them were expressed with 95% CI. In order to make the results of this study more accurate, we adopted the random effect model and the inconsistency model to carry out this study. We first compared all the treatment strategies pairwise and calculated their ORs. For all primary and secondary outcomes, additional contour line funnel plot were used to detect publication bias, and trim and fill method were used to evaluate the stability of the results. Finally, we used the surface under the cumulative ranking curve (SUCRA) to rank the efficacy of the treatment strategy in each outcome. In addition, we did this NMA within a frequentist framework. Funders did not intervene in study design, data collection, data analysis, data interpretation and article writing.

## Results

### Quality and Characteristics of the Included Studies

**FIGURE 1:**
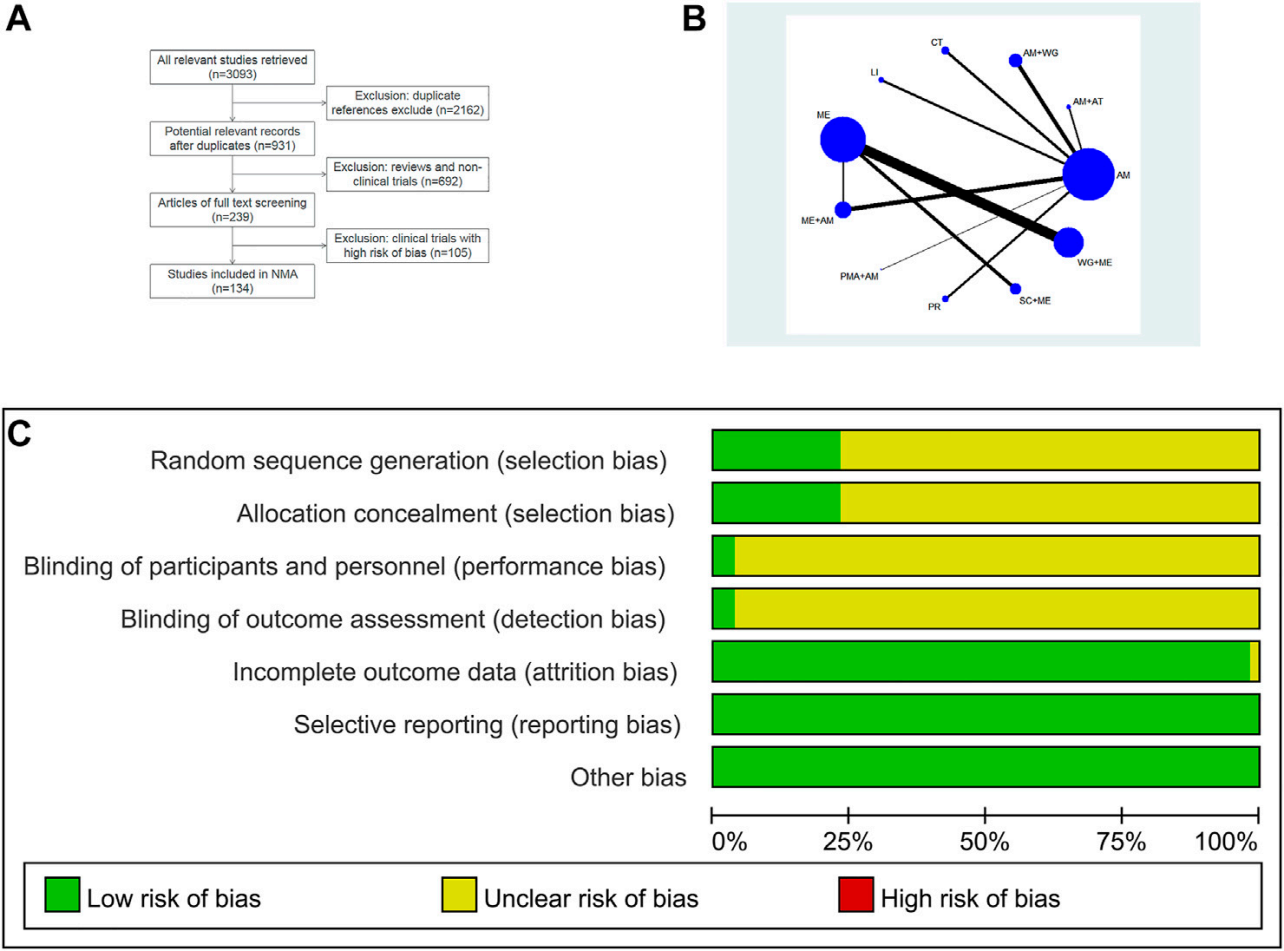
**(A)** Flowchart of studies selection; **(B)** Network of comparisons included in the analysis; **(C)** Methodological quality assessment of the risk of bias for included studies.

A total of 3,093 studies were retrieved through the search strategy. Two thousand one hundred and sixty-two duplicate reports, 83 reviews, 609 animal studies, and 105 clinical studies that did not conform to the randomized control principle were excluded. In the end, 134 studies were included (Figure 1A) (Bai, 2019; Chen and Lin, 2015; Chen and Hu, 2020; Chen, 2019a; Chen et al., 2012; Chen and Wei, 2019; Chen, 2019b; Chen, 2017; Cheng et al., 2019; Dong, 2018a; Dong, 2018b; Duan, 2016; Er, 2020; Fan, 2020; Fan, 2019; Fang, 2018; Feng, 2017a; Feng, 2017b; Feng et al., 2017; Gao, 2018; Gao, 2015; Gao, 2020; Gong and Wan, 2019; Guo, 2018a; Guo, 2018b; Guo and Tai, 2020; Han and Wang, 2014; He et al., 2020; Hou, 2018; Hou, 2019; Hou et al., 2015; Hu and Yang, 2018; Yang, 2018; Yang and Xi, 2020; Ye, 2012; Yi, 2018; Yi, 2019; You, 2017; Yu, 2018; Yu, 2020; Yu et al., 2015; Yuan, 2017; Yue, 2018; Zeng, 2016; Zhang, 2020b; Zhang, 2017b; Zhang, 2018a; Zhang, 2018b; Zhang et al., 2016b; Zhang and Zhang, 2013; Zhang, 2016a; Zhang, 2016b; Zhang, 2019c; Zhang, 2017a; Zhang, 2019a; Zhang, 2019d; Zhang, 2019b; Zhang, 2015; Zhang, 2020a; Zhang et al., 2016a; Zhao and Long, 2017; Zhao et al., 2020; Zhao, 2018; Zhen, 2019; Zhou, 2016a; Zhou, 2016b; Zhou, 2019; Zhou, 2015a; Zhou, 2015b; Zhu, 2019; Zhu, 2018; Zhuang, 2017; Hu, 2016; Huang, 2016; Huang, 2018; Huo, 2019a; Huo, 2019b; Jiang et al., 2017; Jin et al., 2018; Jin, 2019; Ju, 2016; Li, 2020c; Li, 2018a; Li, 2020b; Li et al., 2018; Li, 2020a; Li et al., 2020b; Li, 2020d; Li et al., 2020a; Li, 2018b; Li, 2014; Liang, 2018; Lie, 2018; Liu, 2017; Liu, 2018a; Liu and Wang, 2015; Liu, 2018b; Long, 2019; Luo, 2016; Lv et al., 2020; Meng, 2018; Pan et al., 2017; Peng, 2017; Peng and Liu, 2019; Qian, 2015; Qu, 2016; Ren, 2018; Ren, 2020; Ruan, 2020; Song et al., 2019; Sun and Gu, 2019; Sun, 2018; Sun, 2020; Tan et al., 2020; Wang et al., 2018b; Wang, 2018b; Wang et al., 2018a; Wang, 2013; Wang, 2018a; Wang, 2016; Wang et al., 2020; Wang, 2020; Wang and Qi, 2016; Wang, 2017; Wu, 2016; Xiao et al., 2017; Xu, 2019a; Xu, 2019b; Xu, 2018; Xun, 2017; Xun, 2020; Yang and Zhang, 2019; Yang et al., 2017; Yang, 2017). This study involved a total of 11 treatment strategies (Figure 1B). Among the included studies, 130 studies clearly reported the randomization method adopted, and four studies did not clearly report the randomization method. The data of 134 studies are complete. In addition, three studies clearly reported the type of blinding used. There are no selective reports in all the included studies. We assessed the quality of included studies in accordance with the Cochrane risk-of-bias tool. Each evaluation principle was divided into “high risk,” “low risk,” and “unclear” (Figure 1C). It can be seen that there is no high risk in the included studies. All the studies included in this study were designed in parallel, and the test sites were all in China. All patients were diagnosed as arrhythmia of CHD in regular hospitals according to clear diagnostic criteria. This study involved 13,951 patients. The number of men and women is roughly equal, and the average age is about 57 years old. The basic information of the studies included in this article is listed in Supplementary Table S1.

### Primary Outcome

**FIGURE 2:**
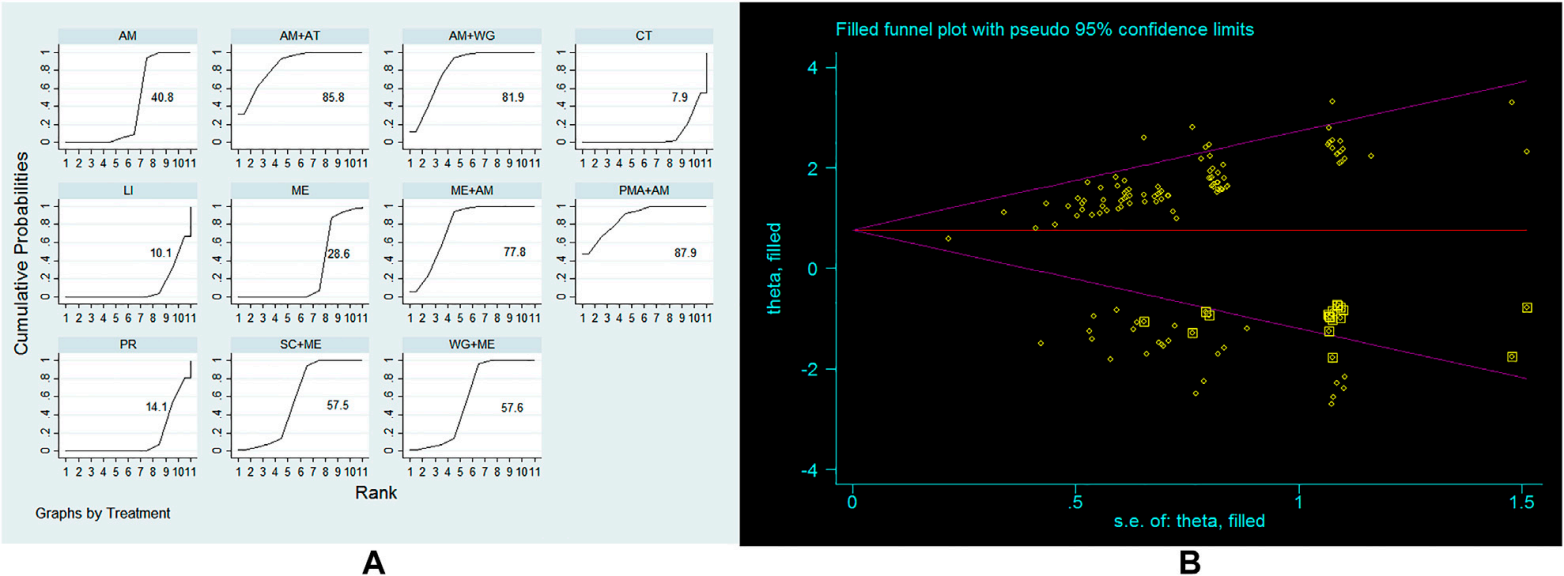
**(A)** Ranking for efficacy of 11 treatment strategies; **(B)** Sensitivity analysis of this study.

The primary outcome indicator of our study is effective rate. And all the included studies reported on this indicator. In order to correct the possible heterogeneity within the study, we decided to use the random effects model to calculate the ORs. We use Stata (13.0) to perform a mixed comparison of all treatment strategies, and obtain the superiority of each treatment strategy compared to other treatment strategies (Supplementary Table S2). Then we draw SUCAR to further comprehensively rank the improvement of the main outcome of these 11 treatment strategies to further explore which treatment strategy is the best (Figure 2A). The results found that PMA+AM, AM+AT, AM+WG are the top three treatment strategies, followed by ME+AM, WG+ME, SC+ME, AM, ME, PR, LI, and CT. Since the combined OR value of each treatment strategy is derived from direct comparison and indirect comparison between treatment strategies, in order to further verify the accuracy of the results of this study, we used trim and fill method to perform a sensitivity analysis on this outcome (Figure 2B). It was found that filling the blank sample did not cause a significant change in the OR value, so the results of this study are stable.

### Secondary Outcome

**FIGURE 3:**
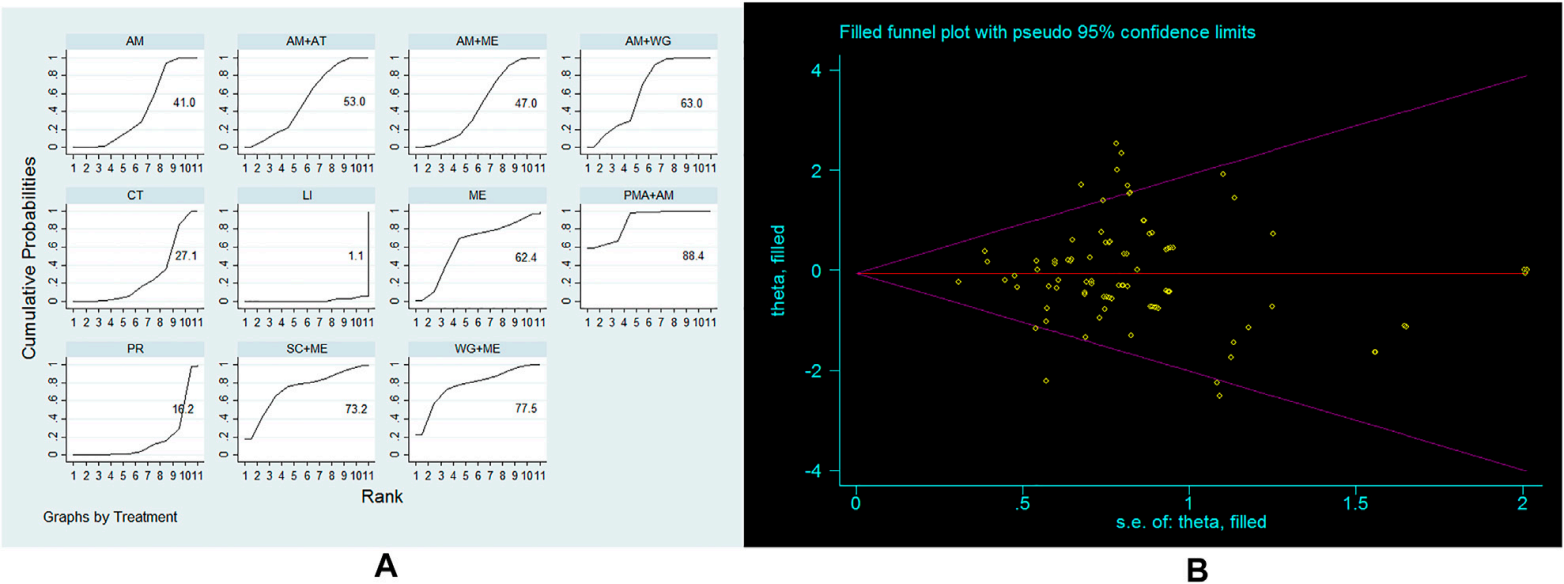
**(A)** Ranking for adverse reaction rate of 11 treatment strategies; **(B)** Sensitivity analysis of this study.

The primary outcome indicator of our study is adverse reaction rate. 88 studies have reported on this indicator, and the main adverse reactions include digestive tract reactions, allergic reactions, hypotension, central nervous system reactions, arrhythmia, abnormal liver function, etc. We use Stata (13.0) to perform a mixed comparison of all treatment strategies, and obtain the superiority of each treatment strategy compared to other treatment strategies (Supplementary Table S3). Then we draw SUCAR to further comprehensively rank the improvement of the secondary outcome of these 11 treatment strategies to further explore which treatment strategy is the best (Figure 3A). We ranked all treatment strategies according to the SUCRA value. The larger the SUCRA value, the lower the incidence of adverse reactions. The results found that PMA+AM, WG+ME, SC+ME are the top three treatment strategies, followed by AM+WG, ME, AM+AT, AM+ME, AM, CT, PR, and LI. Since the combined OR value of each treatment strategy is derived from direct comparison and indirect comparison between treatment strategies, in order to further verify the accuracy of the results of this study, we used trim and fill method to perform a sensitivity analysis on this outcome (Figure 3B). It was found that filling the blank sample did not cause a significant change in the OR value, so the results of this study are stable.

### Bias Analysis

In order to observe whether the results of this study are affected by reporting bias, we draw a additional contour line funnel plot. The results show that most of the studies are located in the upper part of the additional contour line funnel plot, indicating that the dispersion of the samples is low. In addition, the results of the bias test showed that most samples did not have significant reporting bias (*p* > 0.05). Therefore, the results of this study are accurate and stable.

## Discussion

In recent years, studies have shown that more and more patients with coronary heart disease combined with arrhythmia, more than 90% of sudden cardiac deaths are induced by arrhythmia (Yan-Hua, 2018; Dong et al., 2019). However, while the currently commonly used antiarrhythmic drugs improve arrhythmia in patients, there are also many adverse reactions such as promoting arrhythmia. This study conducted a comprehensive evaluation of 11 antiarrhythmic drug therapies frequently used in clinical. All treatment strategies can significantly improve the patient’s arrhythmia, PMA+AM, AM+AT, AM+WG are the top three treatment strategies. In terms of safety, PMA+AM, WG+ME, and SC+ME are the top three treatment strategies. In addition, this study also observed the bias and stability of each outcome, and found no significant bias and instability, which also enhanced the accuracy of the results of this study. However, it should be noted that in the two outcomes of this study, the SUCRA values of the top three treatment strategies are very close. Therefore, although they have significant advantages over other treatment strategies, they do not represent the advantages and disadvantages of the three of them. However, PMA+AM ranks first in the SUCRA value of the two outcome indicators, which seems to indicate that it is the most recommended treatment strategy.

AM as a broad-spectrum antiarrhythmic drug, has the most clinical application at present. It has a good effect on a variety of arrhythmias, but the incidence of adverse reactions is about 80%, and the main adverse reactions include thyroid toxicity, gastrointestinal toxicity, abnormal liver function, etc. (Lavon and Goldman, 2019) PMA can increase myocardial energy supply and reduce myocardial energy consumption. It has a good anti-arrhythmic effect, but there are also adverse reactions such as nausea, vomiting, chest tightness, and vascular irritation pain (Yong, 2013). The combination of the two can significantly improve the incidence of arrhythmias, and also significantly reduce the incidence of adverse reactions such as vomiting, liver function decline and thyroid function decline. This may be because the combination reduces the dose of the drug used alone.

In addition, it is worth mentioning that among the 11 treatment strategies involved in this study, three of them were combined application of chemical drugs and TCM (WG+ME, SC+ME, AM+WG). WG and SC which commonly used anti-arrhythmia drugs in China are included in Chinese pharmacopoeia, and they have definite efficacy and good safety (Shi-Jun, 2019; Shi et al., 2020). This study showed that the anti-arrhythmic efficacy of WG+ME, SC+ME and AM+WG was significantly better than that of AM and ME alone, indicating that TCM could significantly improve the anti-arrhythmic efficiency of chemical drugs, and AM+WG ranked in the top three. From the perspective of drug safety, the safety of WG+ME, SC+ME and AM+WG was also significantly better than that of AM and ME alone. This indicates that the combination of TCM with chemical drugs can significantly reduce the incidence of adverse reactions when chemical drugs are used alone, and WG+ME and SC+ME are the top three in the safety ranking. As a characteristic treatment strategy in China, traditional Chinese medicine can not only significantly improve the efficacy of chemical drugs against arrhythmia, but also have more potential in reducing the incidence of adverse reactions of chemical drugs.

Although there are slight instability and bias in the results of this study and there are limitations in the quality of RCTs, these have reduced the strength of the NMA. However, overall, this NMA still provides clinicians with detailed comparisons of common therapeutic strategies and provides reference for clinical use. The effectiveness and safety of these drugs in the treatment of arrhythmia of CHD need to be further validated in future clinical studies. Several large-scale and high quality randomized uniform criteria are needed to further confirm its effectiveness and safety.

## Conclusion

This article shows that all the 11 treatment strategies concerned in this study can effectively improve arrhythmia of CHD. Among them, PMA+AM, AM+AT, AM+WG have a higher total effective rate, and PMA+AM, WG+ME, SC+ME have a lower incidence of adverse reactions. PMA+AM seems to be the most recommended intervention. In addition, the combination of multiple drugs usually has better efficiency and lower incidence of adverse reactions, especially the combination of TCM and chemical drugs has more potential in improving the effectiveness and safety of anti-arrhythmia. In short, this NMA provides clinicians with a detailed evaluation of the advantages of a variety of common anti-arrhythmic treatment strategies, and provides clinicians with medication references.

## Data Availability

The original contributions presented in the study are included in the article/Supplementary Material, further inquiries can be directed to the corresponding author.
