# Supplementary material for: The Clinical Efficacy and Safety of 11 Commonly Used Treatment Strategies Improving Arrhythmia of CHD in China: A Network Meta-Analysis
Source: Front Pharmacol. 2021 Sep 20;12:741716. doi: 10.3389/fphar.2021.741716 (PMC8488134; doi:10.3389/fphar.2021.741716)
Supplement: Supplementary file 1 [file DataSheet3.docx]

**The botanical drugs and species included in TCM**

**Wenxin Granules (Granules):**

1. Changium smyrnioides Wolff( Apiaceae; CHANGII RADIX );
2. Polygonatum sibiricum F.Delaroche (Asparagaceae; POLYGONATI RHIZOMA );
3. Panax notoginseng (Burk.)F. H. Chen ( Araliaceae; NOTOGINSENG RADIX ET RHIZOMA );
4. Nardostachys jatamansi DC. ( Valerianaceae; NARDOSTACHYOS RADIX ET RHIZOMA );

**Shensong Yangxin Capsules (Capsules):**

1. Panax ginseng C. A. Mey ( Araliaceae; GINSENG RADIX ET RHIZOMA )
2. Ophiopogon japonicus (Thunb.) Ker Gawl. (Asparagaceae; OPHIOPOGONIS RADIX )
3. Cornus officinalis Sieb. et Zucc. ( Cornaceae; CORNIFRUCTUS )
4. Salvia miltiorrhiza Bunge ( Lamiaceae; Salviae miltiorrhizae radix et rhizoma )
5. Ziziphus jujuba Mill. ( Rhamnaceae; ZIZIPHI SPINOSAE SEMEN )
6. Taxillus chinensis (DC.) Danser ( Loranthaceae; TAXILLI HERBA )
7. Paeonia laciflora Pall. ( Ranunculaceae; PAEONIAE RADIX RUBRA )
8. Nardostachys jatamansi (D.Don) DC. (Caprifoliaceae; NARDOSTACHYOS RADIX ET RHIZOMA )
9. Coptis chinensis Franch ( Ranunculaceae; COPTIDIS RHIZOMA )
10. Schisandra sphenanthera Rehd. & E.H.Wilson. ( Magnoliaceae; SCHISANDRAE SPHENANTHERAE FRUCTUS )

**Preparation of chemical drugs:**

1. Amiodarone hydrochloride tablets (tablets);
2. Metoprolol tartrate tablets (tablets);
3. Atenolol tablets (tablets);
4. Ppotassium aspartate and magnesium aspartate injection (injection);
5. Propafenone tablets (tablets);
6. Lidocaine hydrochloride injection (injection).
